# Effect of Diet Supplemented with Nano-Selenium on Reproductive Performance, and Sexual Hormones, Pathological Response of Nile Tilapia (*Oreochromis niloticus*)

**DOI:** 10.3390/ani16081142

**Published:** 2026-04-09

**Authors:** Hualiang Liang, Mingchun Ren, Ahmed Mohamed Aboseif, Enas A. Ramadan, Ramadan M. Abou Zied, Mohamed F. Sadek, Junjie Qin

**Affiliations:** 1 Key Laboratory of Integrated Rice-Fish Farming Ecology, Ministry of Agriculture and Rural Affairs, Freshwater Fisheries Research Center, Chinese Academy of Fishery Sciences, Wuxi 214081, China; 2 National Institute of Oceanography and Fisheries (NIOF), Cairo 11796, Egypt; 3 Faculty of Agriculture, Fayoum University, Fayoum 63514, Egypt; 4 Wuxi Fisheries College, Nanjing Agricultural University, Wuxi 214081, China

**Keywords:** aquaculture, nanotechnology, hematology, testosterone, progesterone

## Abstract

This research aimed to examine the effects of different dietary levels of nano-selenium (NSE) on the reproductive performance, gonad hormones, histopathology, growth performance, feed utilization, and body indices in adult Nile tilapia, *Oreochromis niloticus*, broodstock for 90 days. The initial weights of the fish were 278.6 ± 5.5 (males) and 178.4 ± 1.6 (females). They were distributed randomly to 15 tanks with 20 fish in each tank (15 females and 5 males, sex ratio 3:1), with each treatment conducted with three replicates. The contents of NSE in five isocaloric and isonitrogenous practical feeds were 0 mg/kg (control), 1 mg/kg (T1), 2 mg/kg (T2), 3 mg/kg (T3), and 4 mg/kg (T4). Results show that the final weight (FW), weight gain (WG), specific growth rate (SGR), protein efficiency ratio (PER), and feed conversion ratio (FCR) were significantly better in T2 and T1, followed by the control, in comparison with others. On the other hand, growth efficiency was decreased in T3 and T4 of both males and females. Referring to body indices and reproductive performance, females were higher than males in the hepatosomatic index (HSI), where the lowest treatment was the control and T4 for both males and females. Female Nile tilapia brood fish given NSE improved reproductive performance indicators (egg number, total egg, and fry number) when compared with the control. With increasing levels of NSE in the feed, the levels of testosterone and progesterone hormones were increased. The highest values for testosterone were in T4, followed by T3, then T2, T1, and the control. The same trend was observed across the progesterone treatments. Additionally, the results of histopathological examination indicate differences in tissues between different treatments as a result of the addition of NSE. These results indicate that NSE supplementation at low levels could lead to improved growth and reproductive efficiency of Nile tilapia.

## Simple Summary

The objective of this study was to investigate the influence of different levels of dietary nano-selenium (NSE) on the reproductive performance, gonad hormones, histopathology, growth performance, feed utilization, and body indices of adult Nile tilapia. The addition of NSE at 1 mg/kg and 2 mg/kg significantly improved growth performance metrics such as FW, WG, SGR, PER, and FCR when compared with higher levels and the control. Female Nile tilapia exhibited better reproductive performance indicators, such as egg number and fry number. Increasing NSE levels in the diet corresponded with higher levels of testosterone and progesterone. Histological examinations revealed differences in gonadal tissues among the treatment groups. The study concluded that low levels of NSE can be advantageous for the growth and reproductive performance of Nile tilapia, while also emphasizing the need for balanced dietary formulations to avoid potential negative effects associated with higher selenium (Se) levels.

## 1. Introduction

A rapidly growing business, aquaculture today contributes greatly to the production of water-dwelling species worldwide. Growing fish demand has led to the rise in popularity of intense aquaculture systems, which has raised fish stress levels and exacerbated the onset of diseases, in turn causing large financial losses [1]. The application of nanotechnology and the adoption of sound fishery management techniques are crucial for promoting enhanced yield and variation in aquaculture [2]. Nanotechnology is among the 21st century’s most fascinating new technologies. By observing, measuring, manipulating, integrating, controlling, and manufacturing materials at the nano scale, the concepts of nanoscience can be applied to real-world scenarios [3]. It is becoming more widely acknowledged that nanotechnology is revolutionizing aquaculture by tackling important issues, including feed efficiency, environmental sustainability, and disease control. The aquaculture sector can increase sustainability and productivity while reducing adverse effects on aquatic ecosystems by incorporating nanomaterials and cutting-edge methods.

Nanotechnology in aquaculture enhances fish growth rates and nutrient absorption. Nano-enhanced diets also reduce feed waste and support healthier stocks [4]. The goal of nanotechnology is to produce a range of nanoparticles, such as Se, Cu, Fe, FeO, Zn, and ZnO, that are helpful in physiological uses, particularly the transport of drugs and nutrients. Furthermore, improved mineral absorption, which is necessary for tilapia to develop and thrive, has been connected to the inclusion of nano-selenium (NSE) in diets [5]. Supplementing with nano-selenium has manifested encouraging outcomes in improving the fish’s immune system, growth performance, and general health. NSE is a useful dietary supplement in aquaculture because of the numerous studies that have shown that it can dramatically amplify growth rates, biochemical indices, and immune-related gene expression in Nile tilapia, *Oreochromis niloticus*. Significant increases in weight gain and particular growth rates in Nile tilapia have been found to be associated with NSE supplementation at doses of 1.5 mg/kg [6,7]. According to research, NSE increased overall survival rates and feed conversion ratios, suggesting improved nutrient absorption and digestibility [5,8]. According to Zahran et al. [7], NSE has been demonstrated to upregulate immune-related genes, including interleukin 8 and interleukin 1 beta, which are essential for preserving gut health and immunological defense. According to Al-Wakeel et al. [6], the lack of inflammatory reactions in fish fed NSE indicates a positive influence on their health without any negative side effects.

Although there are clear advantages of using NSE in tilapia diets, it is important to take into account possible long-term consequences and the requirement for additional research to determine the best dosage and safety profiles for aquaculture methods. The impacts of supplementing with dietary nano-selenium on *Cirrhinus mrigala* fingerling growth efficiency, nutrient digestibility, and hematological have been investigated by Ahmad et al. [5]. According to their research, feeding *Cirrhinus mrigala* fingerlings with NSE greatly improves their growth performance. The highest WG and the best FCR were obtained at the optimal dosage of 1 mg/kg. Supplementing with NSE also enhanced hematological indicators.

The farming of species such as the Nile tilapia (*Oreochromis niloticus*), one of the most significant tropical species in global aquaculture due to its rapid growth rate, substantial market size, broad environmental tolerance, and high consumer preference, has been investigated by Mohammady et al. [9].

The effects of different concentrations of NSE, a naturally occurring antioxidant and metabolic regulator, on the growth performance, digestive enzymes, immunological resistance, and antioxidant capacity of *Oreochromis niloticus* exposed to *Aspergillus flavus* infection have been investigated by Eissa et al. [10]. In accordance with the study’s findings, feeding Nile tilapia diets supplemented with NSE greatly increased their growth performance, feed efficiency, and survival rates, especially when the concentration was 1.0 mg/kg diet. Fish challenged with *Aspergillus flavus* had a lower mortality rate when fed diets supplemented with NSE, suggesting that the nanoparticles strengthened the fish’s immune response.

Dietary NSE can positively influence the reproduction of Nile tilapia by increasing fry number, improving gonadal architecture, and enhancing sexual hormones, leading to better spawning performance and survival rate. Ghanem et al. [11] studied the effect of dietary nano-selenium and/or Vitamin E and their interplay on the reproductive physiology of Nile tilapia broodstock. The research findings established that dietary supplementation with 1 mg NSE kg^−1^ and 100 mg VE kg^−1^, particularly in combination, can be effectively utilized to enhance reproductive performance in Nile tilapia broodstock. The study demonstrated that this nutritional strategy provides comprehensive support for reproductive efficiency through multiple physiological pathways, including hormonal regulation, gene expression modulation, and gonadal development optimization.

An earlier study focused mainly on growth, immune, and antioxidant responses in juveniles or on single reproductive endpoints, often at one or two inclusion levels of nano-selenium, without exploring the threshold at which beneficial effects shift towards potential toxicity (selenosis) in brood fish. Therefore, the present study provides novel, integrated evidence on the optimum and upper safe limits (1–2 vs. 3–4 mg/kg, respectively) of nano-selenium for Nile tilapia broodstock, linking productive, endocrine, and tissue-level responses in the same experimental framework. Thus, the objective of this study was to investigate the influence of different levels of dietary NSE on reproductive performance, gonadal hormones, histopathology, growth performance, feed utilization, and body indices in adult Nile tilapia.

## 2. Materials and Methods

Experimental work took place in the fish nutrition lab of the National Institute of Oceanography and Fisheries (Qanater Khaireya), Egypt. The study was carried out between July and September (90 days). All experiments were approved by the authority of the NIOF Committee for Institutional Care of Aquatic Organisms and Experimental Animals (NIOF-AQ4-F-25-R-008).

### 2.1. Diet Preparation

Five isocaloric (22 KJ/g feed), isonitrogenous (35% crude protein) practical diets were utilized (Table 1). Five feeds with different levels of NSE were prepared, with the contents of NSE being 0 mg/kg (control), 1 mg/kg, 2 mg/kg, 3 mg/kg, and 4 mg/kg, in turn marked as C, T1, T2, T3, and T4, respectively. NSE-purchased particles had molecular weights ranging from 15,000 to 20,000 Daltons (Now Foods 395 S, Glen Ellyn Rd, Bloomingdale, IL 60108, USA). Nano-selenium was first dissolved in sterilized distilled water and then sprayed homogeneously onto the feed pellets using a fine mist spray, followed by thorough mixing and drying at 60 °C for 6 h. This protocol is similar to that reported by Eissa et al., Mohtashemipour et al., and Rathore et al. [10,12,13]. Although the current study did not perform a separate physicochemical analysis to quantify selenium stability after drying at 60 °C, the low-temperature drying procedure is consistent with previous practices in aqua feed nano-supplementation, which aim to preserve the integrity of nanoparticles while removing excess moisture.

### 2.2. Description of the Experiment

The study was conducted in fiberglass tanks (with a water volume of 2500 L), provided with fresh, clear well water, and with 20% of the pond water changed daily. The tanks received aeration via an aquarium air pump, which maintains an adequate oxygen level in the experimental tanks. Nile tilapia mix males and females were acquired from a commercial hatchery. Their starting body weights ranged from 278.6 ± 5.5 male and 178.4 ± 1.6 female and they were randomly assigned to 15 tanks, with 20 fish in each tank (15 females and 5 male, sex ratio of 3:1). The treatment was designed in a triplicate replication. Fish were acclimated to experimental conditions for two weeks before feeding. The fish were fed to apparent satiation 6 days per week [10,12,13], three times daily (at 9:00 am, 1:00 pm, and 5:00 pm) throughout the experimental period. Fish were weighed two times per month and bi-weekly following 24 h of starvation; feeding was withheld on the day of weighing. Throughout the experiment, the water temperature fell within the range of 28–30 °C. Dissolved oxygen and temperature were monitored using Professional Plus twice daily at 08:00 am and 3:00 pm. The pH was measured firsthand in the water column of tanks weekly during the experiment using a pH meter (Orion pH meter, Abilene, TX, USA). These conditions are suitable for tilapia rearing, as mentioned in previous research, such as that of Goda et al. [15].

### 2.3. Sample Collection

After the experiment, all of the fish were starved for 24 h. First, the weights of all of the fish in the experimental groups were measured to calculate their growth performance. Then, five fish per tank were anesthetized (MS-222) and weighed for blood collection. The collected blood was then separated by centrifugation to obtain the plasma. Plasma was maintained at 20 °C until used. Finally, the fish was dissected, and the weights of the liver and gonads were measured. Subsequently, the liver and intestines were collected for hematoxylin and eosin (H&E) analysis.

### 2.4. Analyses of Hematological Parameters and Hormonal Evaluation

The caudal veins of three fish for each replicate were used to get blood samples after being anesthetized with 3-aminobenzoic acid ethyl ester (MS 222, 100 mg/L, Sigma, St. Louis, MO, USA). These were then centrifuged at 3000× *g* rpm for 10 min after being left to clot overnight at 4 °C. Enzymes like AST and ALT were measured. Serum was kept at −20 °C until used. Testosterone, progesterone, and estradiol hormones were estimated using HPLC [16]. Hematological parameters were determined according to Reitman and Frankel, Henry, and Tietz [17,18].

### 2.5. Histopathology Analysis

While the fish intestine and liver samples were collected from the different treatments fixed in neutral buffered formalin 10%, the washed tissues were processed through dehydration and clearing steps, then embedded in paraffin. For histological analysis, the paraffin-embedded blocks were sectioned at a thickness of 5 microns and stained with alizarin red stain and hematoxylin and eosin. Using a light microscope (Olympus BX50, Tokyo, Japan), stained slices were inspected. In each group, the stained tissue sections were assessed for alizarin red area percentage and staining intensity. Area % and color intensity were analyzed microscopically in 10 fields per section under a high-power microscopic field (×400). Area % and color intensity were estimated by the color deconvolution image J 1.52 p software (Wayne Rasband, National Institutes of Health (USA)).

### 2.6. Equations

The measurements obtained were used to calculate growth performance, feed utilization, and somatic index: IW, initial weight (g). FW, final weight (g). WG, weight gain (g) = final weight (g) − initial weight (g). FI, feed intake (g) = total feed/fish. FCR, feed conversion ratio = dry feed fed (g)/(final weight (g) − initial weight (g)). PER, protein efficiency ratio = weight gain (g)/(feed intake (g) × crude protein level (%)). SGR, specific growth rate = 100 × [(Ln (final weight (g)) − Ln (initial weight (g)))/days]. HSI: hepatosomatic index = (liver weight/body weight) × 100; GSI: gonadosomatic index = gonad weight/total body weight) × 100.

### 2.7. Statistical Analysis

The experiment was undertaken using a completely randomized design and, at the end of the experiment, the data collected were subjected to one-way analysis of variance (ANOVA) using statistical software (SPSS 21) to detect significant differences in all parameters. Duncan’s new multiple range tests were used to detect individual differences between treatment means [19]. All data were presented as means ± S.D and a rejection level of *p* > 0.05 would be used for all statistical analyses.

## 3. Results

### 3.1. Growth Performance

Data in Table 2 illustrated the growth performance parameters of the fish groups. The final weight, weight gain, SGR%, PER, and FCR were significantly higher in the 2 mg/kg (T2) and 1 mg/kg (T1) groups, followed by the control group, compared with other groups. Growth and feed utilization efficiency results were equal in male and female groups. On the other hand, growth efficiency decreased in groups of both males and females that were fed higher levels of NSE in their diets (T3 and T4). Significantly, increased growth performance parameter was acquired in fish fed with 2 mg NSE, 1 mg NSE, and the control by comparison to the other groups (*p* < 0.05).

### 3.2. Reproduction Performance

The results in Table 3 indicate that females were found to be higher in HSI than males when given treatments that included 2 mg/kg NSE in their diets, followed by treatments T3, T1, T2, T4 (males) and treatments T3, T4, T1 (females), and that there were no significant differences with the control treatment for both males and females. Additionally, treatment T2 was found to be highest in gonadosomatic index (GSI) for both males and females, without significant differences between treatment T1 (females) and T3 (males). The lowest treatment was the control and the T4 treatment for both males and females.

### 3.3. Gonad Hormones

**Figure 1:**
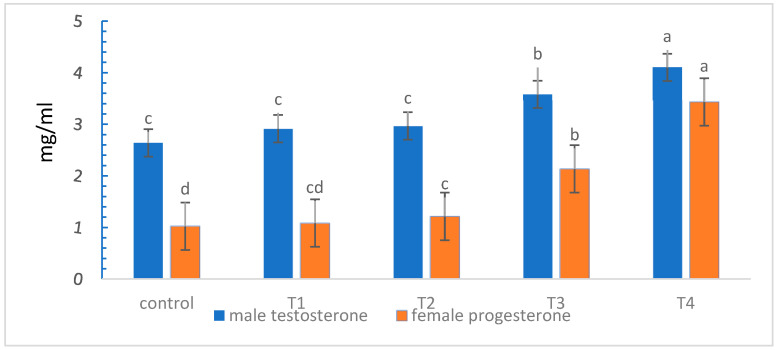
Effect of dietary nano-selenium on gonad hormones for broodstock Nile tilapia *Oreochromis niloticus*. Different superscripts differ significantly (*p* < 0.05).

The effect of dietary NSE on gonad hormones is illustrated in Figure 1. For testosterone, no significant differences were recorded between the control, T1, and T2, while significant differences were observed between T3 and T4. The highest values for testosterone were in treatment T4 (4.11), followed by T3 (3.58), then T2 (2.97), T1 (2.92), and finally the control treatment (2.64). Significant differences were observed between many treatments for progesterone hormone and the values are as follows: (T4 = 3.44, T3 = 2.14, T2 = 1.22, T1 = 1.09, and basal diet = 1.03).

### 3.4. Role of Nano-Selenium on Liver Function and Other Immunological Parameters

**Figure 2:**
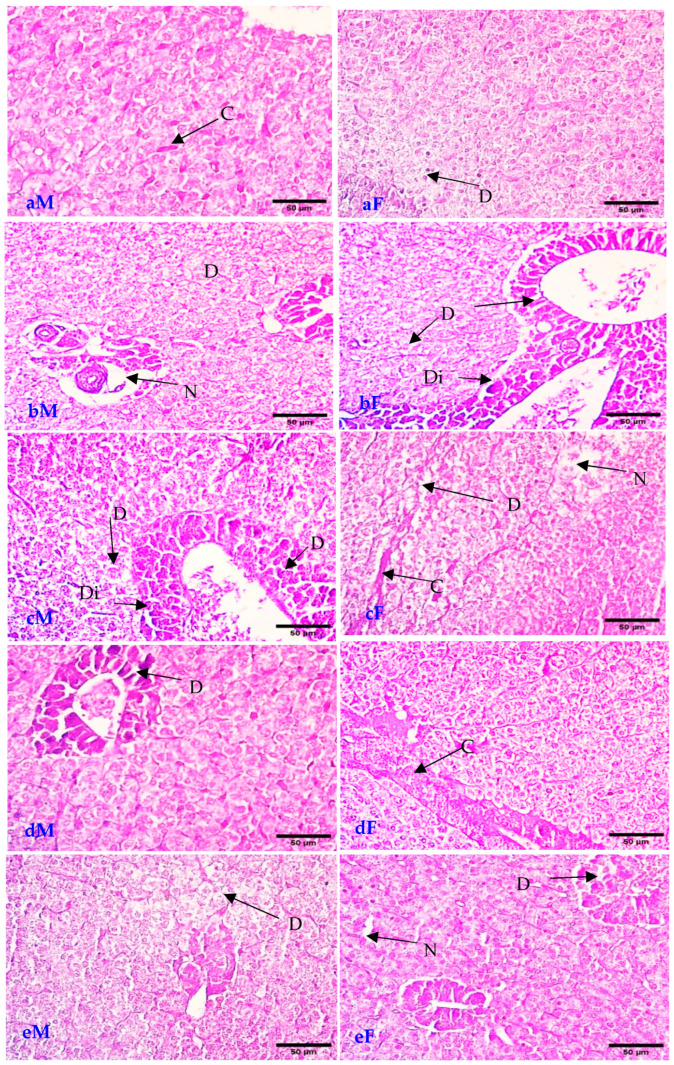
Sagittal histological sections of Nile tilapia *Oreochromis niloticus* male and female in liver. ((**aM**): male and (**aF**): female); the (C) Control group ((**bM**): male and (**bF**): female), the T1 group ((**cM**): male and (**cF**): female), the T2 group ((**dM**): male and (**dF**): female), T3 group ((**eM**): male and (**eF**): female), and T4 group (D) hepatic cells; (N) the hepatic pancreatic structure; and (Di) hepatic vein and degeneration.

**Figure 3:**
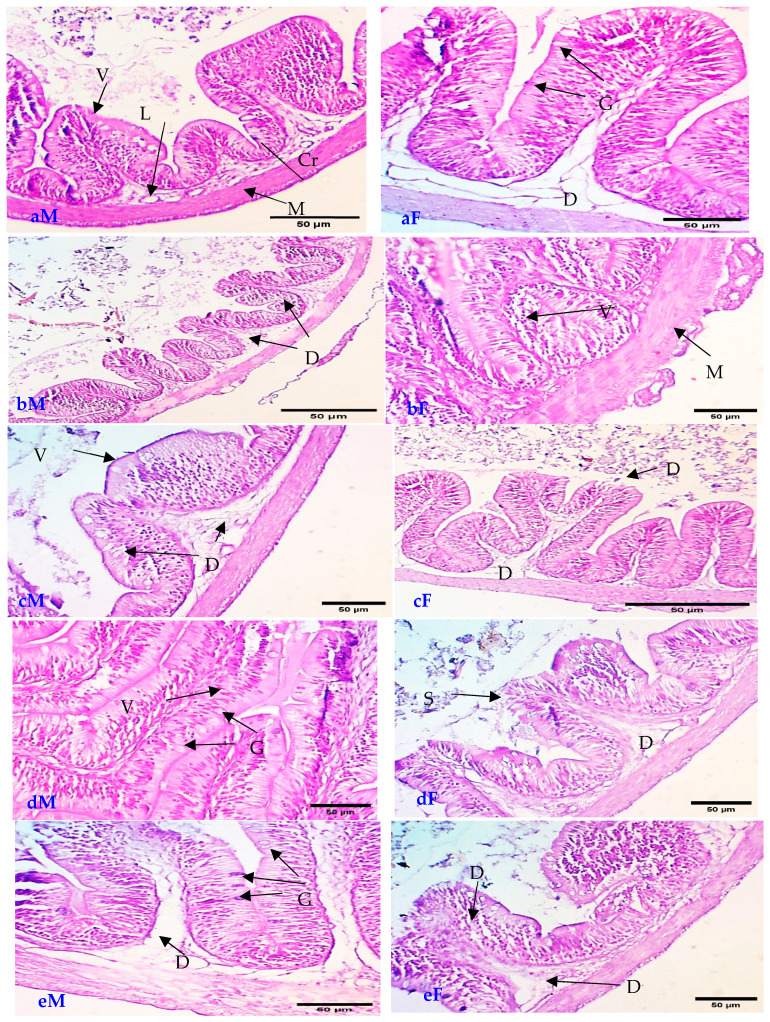
Sagittal histological sections of Nile tilapia *Oreochromis niloticus* male and female intestine ((**aM**): male and (**aF**): female). (C) Control group, ((**bM**): male and (**bF**): female). The T1 group, ((**cM**): male and (**cF**): female). T2 group ((**dM**): male and (**dF**): female), T3 group, ((**eM**): male and (**eF**): female) and T4 group (D) hepatic cells. (N) Hepatic pancreatic structure. (Di) Hepatic vein and degeneration. (G) Slight degeneration of the lamina propria, (V) intestinal villi, and (M) muscular layer.

According to the data supplied, the inclusion of NSE in the diet of Nile tilapia has demonstrated notable impacts on liver function and other health markers as follows (Table 4). Liver enzymes (ALT and AST): The table presents values for alanine aminotransferase (ALT) and aspartate aminotransferase (AST) across different treatment groups. For males, ALT levels decreased from 41.1 (control) to 20.77 (T4), while AST levels also showed a decline from 41.15 to 27.84 as the NSE concentration increased. This suggests that higher levels of NSE may lead to improved liver function, as lower enzyme levels are often associated with better liver health. In females, ALT and AST values also decreased from 36 and 40 (control) to 20.5 and 25.2 (T4), respectively, indicating a similar trend of improved liver function with increased NSE supplementation. Lysosomal Activity: The lysosomal enzyme activity, measured as Lysos, showed a decrease in both genders with increasing NSE levels. For males, this ranged from 444 (control) to 339 (T2) and 369.6 (T4), while for females, it decreased from 271 (control) to 313 (T3). This reduction may indicate a decrease in cellular stress or damage, suggesting that NSE could enhance cellular health. Immunological Parameters: Immunoglobulin M (IgM) levels and lysozyme activity were also assessed. In males, IgM levels increased from 1.95 (control) to 3.79 (T3), while in females, they increased from 2.55 (control) to 3.8 (T3). This increase in IgM suggests enhanced immune response, which is beneficial for overall health and disease resistance. Lysozyme activity, an important marker of innate immunity, also showed an increase in both genders, indicating that NSE may bolster the immune system. Glutathione (GSH) Levels: GSH levels, an important antioxidant, were recorded, showing a slight variation across treatments. For males, GSH levels decreased from 18.9 (control) to 19.2 (T4), while females showed a decrease from 14.26 (control) to 21 (T4). Lysozyme (LZM) levels, an important part of immunity, were recorded, showing a slight variation across treatments. For males, LZM levels decreased from 27.5 (control) to 51.96 (T4), while females showed a decrease from 25 (control) to 40.25 (T4). While the groups received a diet containing NSE, particularly at diminished inclusion levels (T1), they had essentially expanded villi length, width in the intestine, and normal tissue in the liver (Figure 2 and Figure 3).

Figure 2 presents sagittal histological sections of male and female livers from treatment groups supplemented with varying doses of nano-selenium (0, T1, T2, T3, and T4 mg/kg feed). Histological sagittal section of the female liver indicates that (aM) the control group underwent congestion (C) of blood sinusoids. (bM) The T1 group revealed nearly normal tissue with slightly degenerated (D) hepatic cells. (cM) The T2 group revealed degeneration (D) of hepatic tissue and necrosis (N) of hepatic pancreatic structure. (dM) The T3 group revealed dilation (Di) of the hepatic vein and degeneration (D) of the hepatic tissue and pancreatic structure. (eM) The T4 group revealed dilation (Di) of the hepatic vein and degeneration (D) of hepatic tissue and pancreatic structure. Histological sagittal section of the female liver indicated that the (aF) group fed 0 mg nano-selenium/kg feed underwent degeneration (D), necrosis (N) of hepatic tissue, and congestion (C) of blood sinusoids. (bF) The T1 group revealed normal hepatic tissue with slight degeneration (D) of pancreatic structure. (cF) The T2 group revealed congestion (C) of blood vessels. (dF) The T3 group revealed slight degeneration (D) of hepatic tissue and pancreatic structure. (eF) The T4 group revealed degeneration (D) of the pancreatic structure and a small area of necrosis (N) in hepatic tissue.

Figure 3 presents a histological section of the male intestine (aM) control group fed 0 mg nano-selenium/kg feed, showing (V) intestinal villi, (L) lamina propria, (M) muscular layer, and (Cr) intestinal crypt. (bF) The T1 group revealed an increase in the number of goblet cells (G) and slight degeneration in the lamina propria (D). (cF) The T2 group revealed degeneration (D) of the villi tissue and lamina propria. (dF) The T3 group revealed nearly intact tissue of the villi (V) and muscular layer (M). (eF) The T4 group revealed degeneration (D) of villi tissue and lamina propria. Histological section of the female intestine shows (aF) that the control group fed 0 mg nano-selenium/kg feed underwent degeneration (D) of villi tissue and lamina propria. (bF) The T1 group revealed normal villus tissue (V) and increased goblet cells (G). (cF) The T2 group revealed degeneration (D) of the lamina propria and sloughing of the villus tip (S). (dF) The T3 group revealed increased goblet cells (G) with slight degeneration (D) of the lamina propria. (eF) The T4 group revealed degeneration (D) of the villi tissue and lamina propria.

Specimens within the T1 cohort demonstrated an increase in villi dimensions, an elevation in goblet cell density, and a hepatic architecture that closely resembled normalcy, which signifies an improvement in both digestive and absorptive capabilities. Conversely, histopathological evaluations indicated that elevated levels of supplementation (T2:T4) precipitated deleterious modifications, including the degeneration of villi and lamina propria, alongside sporadic necrosis and sloughing within the intestinal tissues of both male and female specimens. In a similar vein, certain hepatic tissues exhibited manifestations of degeneration and vascular dilation at higher concentrations of NSE. These observations imply a dose-dependent influence of nano-selenium, whereby moderate supplementation enhances the integrity and functionality of tissues, while excessive concentrations may inflict cytotoxic repercussions or stress-related responses on intestinal and hepatic tissues. This trend underscores the necessity of refining NSE inclusion levels to attain advantageous outcomes without instigating histological impairment.

## 4. Discussion

### 4.1. Growth Performance

Moges et al. [20] investigated how NSE affected the nutritional value and growth efficiency of Nile tilapia, or *Oreochromis niloticus*. They employed doses of 0.5 mg/kg, 1 mg/kg, and 2 mg/kg. It has been demonstrated that supplementing broodstock Nile tilapia with NSE at a dosage of 1 mg/kg enhances their reproductive performance. This dietary supplement improves the meat quality, growth performance, and immunological and physiological responses of the fish. Empirical studies have established that adding NSE to fish diets can improve growth indicators like weight increase, feeding rates, and total fish size. It can also raise the feed conversion ratio, which gauges how well feed promotes growth. As a result, fish can gain more weight for each unit of feed they consume. In the gastrointestinal system of fish, NSE may improve nutrient absorption. By increasing the bioavailability of selenium and maybe other minerals, the nanoparticles can promote more effective digestion and utilization [21]. Fish nourished with diets enriched with NSE have shown notable increases in weight gain, according to several studies. For example, compared with the control group, which was deprived of selenium supplementation, Nile tilapia fed a diet containing NSE at particular doses (usually about 0.5 to 1 mg/kg) showed greater weight gain [22]. In addition to gaining weight, fish fed NSE have also been shown to grow longer, which suggests improved growth performance overall. In certain instances, fish with NSE in their diet had higher survival rates, which were ascribed to increased immunological and general health. Fish in better health can develop more efficiently and exhibit lower rates of disease or environmental stressor-related mortality [23,24].

NSE has been used by numerous researchers to encourage fish growth. By improving circulation and boosting food absorption into the bloodstream, NSE aids in the growth of fish [25]. The efficiency of these particles is attributed to their nano dimensions, which ensure a large surface area relative to volume [26]. NSE supplementation led to increased intestinal villi length and better feed efficiency, which may have been caused by better intestinal integrity. The capacity of selenium to enhance fish feed utilization can be linked to its function in preserving intestinal integrity, which is accomplished by expanding the intestinal surface area and encouraging the release of brush border enzymes, finally enabling effective nutrient absorption [27]. Subsequent findings have indicated that adding NSE to rats’ diets enhances gastrointestinal function [28].

A study conducted by Ahmad et al. [5] investigated the implications of adding NSE to diets based on sunflower meal (SM) on digestibility, growth performance, and mineral absorption of *Catla catla* fish. Significant increases in mean weight gain, an exceptional average weight gain percentage, a 100% survival rate, and the greatest specific growth rate were among the noteworthy outcomes of the test feed supplemented with 1.5 mg/kg^−1^ NSE. Furthermore, NSE at 1.5 mg/kg^−1^ showed excellent nutritional digestibility. The results of feeding adult Nile tilapia with NSE have been inconsistent. Although some research indicates that NSE at a dosage of 1 mg/kg can improve meat quality and growth performance, other studies show detrimental effects on feed utilization and growth at varying doses. As a result, the dosage may determine how successful NSE is as a dietary supplement overall. The recommended dosage of NSE that is advised for fish varies based on the species and the supplementing goal. Research indicates that a dosage of roughly 0.5 to 2.0 mg/kg of feed is beneficial for Nile tilapia. A typical effective dosage identified in studies is approximately 1 mg/kg of feed, which has been linked to better reproductive and growth effects. To boost growth and immune-related gene expression, a prior study assessed the ideal dietary Se requirement of tilapia at 1.23 mg NSE/kg feed for 90 days [22]. This study’s Se concentrations support prior accounts, with the ideal concentration being between 1.0 and 2.0 mg/kg of Se. This was determined to have a positive effect on tilapia and prevent any negative effects from greater inclusion of Se levels in Nile tilapia. Although Se has advantages when taken in moderation, excessive supplementation might be harmful. As a result, it is critical to measure carefully and follow dosage recommendations [29]. The results related to growth in this research are consistent with the results obtained by many researchers in this regard [5,6,7,10,20,22,30].

### 4.2. Reproduction Performance

A previous study demonstrated the effect of varying levels of the NSE enzyme on body parameters, particularly the GSI and HSI indices of Nile tilapia, showing that adding this enzyme to the diet can significantly improve these parameters. Generally, a higher HSI index is associated with moderate NSE intake, indicating improved liver function and the body’s ability to store nutrients. According to studies, fish growth, feed utilization, and reproductive success are all improved by optimal levels of NSE. This leads to favorable changes in HSI and GSI values, which indicate the fish’s nutritional and overall health. Higher GSI values have been associated with moderate NSE supplementation, suggesting enhanced fish health and reproductive function. Better holistic health, growth performance, and reproductive success of Nile tilapia can be indicated by improvements in HSI and GSI with NSE supplementation. This can result in increased spawning success and healthier fry [20,31,32]. Regarding the number of eggs, and consequently the number of fry, the results indicate an increase in both for all treatments that included NSE. T3 had the highest number of eggs produced and the highest number of fry resulting from hatching these eggs, followed by treatments T1 and T2 (without significant differences between them), then the control treatment, which recorded the lowest number of eggs and resulting fry. Diet has a major impact on Nile tilapia reproductive function in several ways. Gonadal development and general health are supported by a balanced diet that contains vital elements such as lipids, vitamins, proteins, and minerals. For successful reproduction, the proper ratio of fatty acids, particularly omega-3 and omega-6, is essential. By strengthening immunological responses, lowering stress levels, and fostering general health, the addition of particular additives can improve reproductive outcomes. These fatty acids also contribute to hormone production and have an impact on spawning frequency and egg quality [33,34,35,36].

Larval survival and hatching rates were positively impacted by optimal selenium levels in female fish, while improvements in fry metrics and egg quality also suggest that larval survival was likely to be improved. According to certain researchers, male reproductive efficiency may be enhanced by NSE [37]. As stated by Shi et al. [38], supplementing with NSE was found to increase the testis selenium content and testicular and semen GSH-Px activities, protect the integrity of the membrane system, and tighten the organization of the mitochondrial midpiece. Additionally, spermatogenesis, healthy testicular development, the motility, and function of spermatozoa all depend on Se. Because it is a part of the mitochondrial capsula protein and is required for the proper development of spermatozoa, selenium is one of the most crucial components in male reproduction. This has been reported in a study led by Naiel et al. [36], which undertook to assess the impact of different selenium sources in the diet on the reproductive efficiency of red tilapia broodstock. Their findings showed that dietary NSE led to an increase in gonad size, earlier spawning, and higher egg output in terms of spawning performance. Incorporating NSE into the diets of red tilapia broodstock could serve as a reliable and effective method by which to enhance reproductive performance and fry yield, as evidenced by the improved reproductive traits and hormone levels. Fish fed NSE had significantly better spawning indicators than those on sodium selenite or control diets, demonstrating the effectiveness of NSE in enhancing reproductive performance.

The results demonstrate the positive impacts of NSE on reproductive features by showing that all egg biometric indicators, such as weight and diameter, significantly improved in fish receiving NSE. Further evidence of the beneficial effects of NSE on egg quality was provided by the observation that no morphologically aberrant eggs were found in the brood fish fed diets based on selenium. Nutrients primarily stimulate egg formation during fish maturation and spawning and red tilapia that are fed specific diets exhibit larger bodies and organs. Better reproductive health and energy storage for egg development have been indicated by the work of Naiel et al. [36]. In research by Pereira et al. [39], fingerlings from broodstock fed 0.50 mg of organic Se showed better weight gain than fish on the control diet. Diets with incorporated 2–4 mg of NSE/kg have been shown in numerous studies to improve fry survival, hatching success, fertilization rates, and egg quality. In particular, one study found that diets high in selenium increased the weight of the fry and increased the quantity of viable fry generated. According to this, consuming NSE through food may have a beneficial effect on reproductive outcomes [40,41,42,43]. Studies that evaluated various concentrations of NSE (0, 0.5, and 1.0 mg/kg) discovered that, although growth metrics improved, there were no appreciable differences in reproductive parameters across treatments [31]. However, there are conflicting findings. According to one study, supplementing broodstock with NSE did not significantly increase their reproductive performance, indicating that, although it may improve general health, it has no direct effect on egg production [44].

### 4.3. Gonad Hormones

Gonadal hormones can be considerably impacted by the presence of NSE in tilapia diets. Research shows that supplementing with selenium, especially NSE, can increase the activity of reproductive hormones. Reproductive hormones are essential for spawning and general reproductive health, and selenium may have an impact on their synthesis and control. Early-life survival rates and better embryo development have been associated with selenium. According to Penglase et al. [45], selenium facilitates critical activities during early development and fertilization. The decrease in stress indicators and improvement in general health metrics imply that NSE may have a beneficial effect on the hormonal environment, which may have an indirect effect on gonad hormones [7,46].

### 4.4. Histopathology and Biochemical Analyses

The present investigation elucidated that the cohorts subjected to a diet enriched with NSE, particularly at diminished inclusion levels, exhibited an augmentation in both the length and width of intestinal villi and preserved normalcy in hepatic tissue. Within these cohorts, the structural architecture and morphology were nearly representative of baseline conditions; these enhanced morphometric attributes elucidate the advancement of both intestinal and hepatic architecture in the Nile tilapia broodstock that were administered this fortified feed.

The research undertaken by Eissa et al. [10] elucidated that Nile tilapia broodstock subjected to dietary regimens augmented with NSE, particularly at reduced inclusion rates, exhibited markedly enhanced intestinal villi dimensions in both length and width, in conjunction with the maintenance of typical hepatic tissue structure. These augmented morphometric characteristics signify an improvement in both intestinal and hepatic morphology, closely mirroring baseline control conditions.

The results of the current study are also similar to those of studies conducted by Bazina et al. [47], whose results concluded that supplementing diets with NSE and/or VE enhances growth, body composition, biochemical parameters, and histopathology in Nile tilapia. The tilapia study using 0.5, 1, and 2 mg/kg NSE in fingerlings found that 1 mg/kg produced the best growth, while 2 mg/kg markedly reduced relative growth rate and specific growth rate, indicating negative effects of the higher dose and supporting the idea of a dose-dependent toxicity threshold. Similar nonlinear responses, with optimal growth at ~0.8–1 mg/kg and decline or hepatic alterations at higher levels, are reported in other fish species [10,48,49]. A selenium nano vaccine against Streptococcus pyogenes in Nile tilapia improved hematology, antioxidant enzymes, and liver histology, reducing necrosis and inflammation after bacterial challenge, though the relevant study was focused on systemic and hepatic responses, not intestinal morphology [49,50,51]. By contrast, multiple dietary NSE studies in tilapia show combined gut–liver benefits, with improved villus length/width, goblet cells, digestive enzymes, and hepatopancreatic histology. A study on grass carp under high-fat diets showed NSE reversing villi atrophy and tight junction damage, supporting our intestinal improvements, but in a hyperlipidemic context rather than broodstock reproduction. Selenium nano-vaccines improved hepatocyte arrangement and reduced necrosis post-Streptococcus infection in tilapia but lacked intestinal focus, highlighting the superiority of dietary NSE in moderate doses for dual gut–liver protection in our reproductive trial. The consistent preservation of intestinal and hepatic morphology across these studies at optimal low NSE doses (0.5–1.5 mg/kg) reflects its superior bioavailability and antioxidant efficacy via selenoprotein upregulation, outperforming inorganic forms and preventing oxidative disruptions common in aquaculture stressors [35].

Despite the emerging health benefits of NSE, which promote growth and act as an antioxidant at low nutrient levels (typically between 0.3 and 1.5 mg/kg), increasing levels beyond the critical threshold (which is often more than 3–5 mg/kg in most fish species) leads to toxic effects commonly referred to as selenium toxicity (selenosis). This induces antioxidant defense enzymes such as SOD and GPx, resulting in depletion of GSH stores, increased lipid peroxidation (MDA levels), and subsequent cellular damage [10,47]. At the histological level, high doses of selenium have been shown to cause structural damage and functional impairment in vital organs; in this case, focal necrosis (FNC) is indicative of tissue damage. Vacuolation and hematoma were observed, with elevated serum liver enzyme levels (AST, ALT), indicating hepatocellular damage. Signs of angiogenesis and cellular necrosis were also present, while the gills exhibited hyperplasia and lamellar fusion, implying impaired gaseous and osmotic exchange [48]. Furthermore, excessive selenium exposure may impair cellular proliferation and lead to teratogenic effects in advanced stages [49]. It is noteworthy that, although nano-selenium particles possess a wider safety margin and lower toxicity compared with inorganic selenium (e.g., sodium selenite), the risk of their accumulation in muscle tissues remains under uncontrolled usage. This necessitates adherence to optimal dosage levels to avoid potential environmental and health risks [49].

The current study outcomes are in line with those of Surai [50], who discovered that incorporating NSE increased the number and activity of gut microorganisms as well as the potency of digesting proteases. Additionally, adding NSE to the meals increased the number of goblet cells and the length/width of the villus in the fish intestine, along with increased protein synthesis and cell proliferation, all of which improved the alimentary canal’s surface absorption area. Se serves as a co-enzyme factor that is essential for the activation of the digestive enzymes and gut flora, which changes the guts architecture and absorption capacity and ultimately enhances animal performance and feed efficiency [10,52]. The results are also consistent with recent studies conducted by [53].

### 4.5. Immunological Responses

Previous research has shown results that align with the findings regarding the effects of NSE on liver function and overall health in Nile tilapia. Following are some key studies that support these results: A study by Zhang et al. [54] demonstrated that an NSE-enriched diet significantly reduced ALT and AST levels in fish, indicating improved liver function. This is consistent with the findings in the current study, where both male and female Nile tilapia showed decreased liver enzyme levels with increased NSE supplementation. NSE improved the antioxidant capacity of the fish, which raised GSH levels, according to Eissa et al. [10]. The general trend of enhanced antioxidant status is consistent with Lu et al.’s findings [55], indicating that NSE can help reduce oxidative stress in aquatic species, even if the current investigation revealed minor alterations in GSH levels. NSE is a potent antioxidant that helps the body fight off free radicals. This mechanism reduces oxidative stress, which can damage cells and tissues. The current study’s findings indicate that the reduction in oxidative stress is primarily responsible for the improvement in liver enzyme levels and overall health observed in Nile tilapia. The recent findings of improved immunological parameters in Nile tilapia are supported by a study by Li et al. [56], which found that supplementing fish with NSE raised immunoglobulin levels and lysozyme activity. Both male and female tilapia in the current study showed higher IgM levels, which is in line with the findings of Li et al. [56] and suggests that NSE has elevated the immunological response.

The importance of selenium for the immune response is widely known. NSE can increase the generation of immunoglobulins and immune cell activity, which are essential for fighting infections. The increase in immunological indicators, such as IgM levels, in Nile tilapia may be explained by the immunomodulatory effects of NSE. According to Wang et al. [57], NSE may lower lysosomal enzyme activity, indicating less cellular stress and better cellular health. The current study supports the idea that NSE improves cellular function in fish by showing that lysosome activity decreased as levels of NSE increased. According to Fan et al. and Aboseif et al. [58,59], supplementing with NSE has a good impact on the health and growth performance of various fish species.

## 5. Conclusions

The addition of NSE at 1 mg/kg and 2 mg/kg significantly improved growth performance metrics such as FW, WG, SGR, PER, and FCR when compared with higher levels and the control. Female Nile tilapia exhibited better reproductive performance indicators, such as egg number and fry number. Increasing NSE levels in the diet corresponded with higher levels of testosterone and progesterone. Histological examinations revealed differences in gonadal tissues among the treatment groups. The study concluded that low levels of NSE can be advantageous for the growth and reproductive performance of Nile tilapia, while also emphasizing the need for balanced dietary formulations to avoid potential negative effects associated with higher Se levels. It is also recommended to study higher concentrations of NSE to determine the maximum levels of NSE use in the diets of Nile tilapia broodstock.

## Figures and Tables

**Table 1:** Ingredients and chemical composition of the basal diet.

Ingredients	Level (%)
Fish meal	20
Soybean meal	30
Yellow corn	28
Wheat bran	6
Corn gluten	5
Molasses	1
Starch	3
Soy oil	4
Vitamin and mineral mixture *	1.5
Dried garlic	0.5
Dicalcium phosphate	1
Chemical analysis	
Metabolic energy (kcal) **	22 KJ/g
Crude protein (%)	35
Ether extract (%)	4.7
Total ash (%)	7.8
Crude fiber (%)	3.7

* Vitamin and mineral mixture: Each 1 kg of mixture contains the following: Retinyl acetate: 3000 IU; cholecalciferol: 2400 IU; all-rac-α-tocopheryl acetate: 60 IU; menadione sodium bisulfite: 1.2 mg; ascorbic acid monophosphate (49% ascorbic acid): 120 mg; cyanocobalamine: 0.024 mg; d-biotin: 0.168 mg; choline chloride: 1200 mg; folic acid: 1.2 mg; niacin: 12 mg; d-calcium pantothenate: 26 mg; pyridoxine. HCl: 6 mg; riboflavin: 7.2 mg; thiamin. HCl: 1.2 mg; sodium chloride (NaCl: 39% Na, 61% Cl): 3077 mg; ferrous sulfate (FeSO4·7H_2_O, 20% Fe): 65 mg; manganese sulfate (MnSO_4_, 36% Mn): 89 mg; zinc sulfate (ZnSO_4_·7H_2_O, 40% Zn): 150 mg; copper sulfate (CuSO_4_.5H_2_O, 25% Cu): 28 mg; potassium iodide (KI, 24% K, 76% I): 11 mg. ** Metabolic energy (KJ/g) was calculated using the physiological values, CP × 23.9 + lipid × 39.8 + carbohydrates × 17.6 [14].

**Table 2:** Effects of nano-Se on the growth efficiency of broodstock Nile tilapia *Oreochromis niloticus*.

Treatments	IW	FW	WG	FI	FCR	PER	SGR
Male							
C	281.44 ± 1.70	400.30 ± 4.90 ^a^	118.85 ± 0.77 ^a^	285.88 ± 0.15	2.40 ± 0.00 ^b^	1.53 ± 0.00 ^a^	0.39 ± 0.00 ^a^
T1	273.10 ± 1.98	394.22 ± 2.77 ^a^	121.11 ± 1.93 ^a^	282.51 ± 1.19	2.33 ± 0.02 ^b^	1.58 ± 0.01 ^a^	0.41 ± 0.00 ^a^
T2	280.93 ± 3.63	405.55 ± 4.05 ^a^	124.62 ± 1.48 ^a^	286.38 ± 0.52	2.29 ± 0.03 ^b^	1.61 ± 0.02 ^a^	0.41 ± 0.00 ^a^
T3	281.19 ± 1.85	319.72 ± 5.12 ^b^	38.52 ± 3.81 ^b^	284.32 ± 2.01	7.70 ± 1.61 ^a^	0.50 ± 0.05 ^b^	0.14 ± 0.02 ^b^
T4	278.17 ± 2.67	312.59 ± 2.10 ^b^	34.41 ± 3.99 ^b^	286.49 ± 1.16	8.44 ± 1.01 ^a^	0.44 ± 0.04 ^b^	0.13 ± 0.01 ^b^
*p* Value	0.815	0.021	0.022	0.932	0.014	0.002	0.001
Female							
C	178.84 ± 1.55	237.16 ± 1.96 ^a^	58.70 ± 0.41 ^a^	285.88 ± 0.15	4.87 ± 0.05 ^b^	0.76 ± 0.00 ^a^	0.32 ± 0.00 ^a^
T1	178.02 ± 0.94	247.72 ± 1.41 ^a^	69.69 ± 1.08 ^a^	282.51 ± 1.19	4.09 ± 0.61 ^b^	0.91 ± 0.13 ^a^	0.36 ± 0.04 ^a^
T2	179.10 ± 0.50	238.66 ± 1.55 ^a^	59.55 ± 2.05 ^a^	286.38 ± 0.52	4.84 ± 0.59 ^b^	0.77 ± 0.09 ^a^	0.32 ± 0.03 ^a^
T3	177.05 ± 2.36	202.10 ± 2.56 ^b^	25.05 ± 2.28 ^b^	284.32 ± 2.01	11.43 ± 1.35 ^a^	0.33 ± 0.03 ^b^	0.15 ± 0.01 ^b^
T4	180.12 ± 0.48	204.44 ± 1.47 ^b^	24.32 ± 0.56 ^b^	286.49 ± 1.16	11.78 ± 0.22 ^a^	0.31 ± 0.00 ^b^	0.14 ± 0.00 ^b^
*p* Value	0.924	0.019	0.02	0.712	0.001	0.0023	0.032

Note: Data are presented as mean ± SD. Different superscripts within same column differ significantly (*p* < 0.05). Where treatments of males were compared with each other, and treatments of females were compared with each other.

**Table 3:** Impact of varying concentrations of nano-Se on the body indices and reproductive performance of adult Nile tilapia *Oreochromis niloticus*.

Treatments	HSI		GSI		Reproduction Performance		
	Male	Female	Male	Female	Egg number	Fry number	Total egg and fry number
C	2.07 ± 0.24 ^a^	2.58 ± 0.04 ^a^	0.33 ± 0.12 ^b^	5.23 ± 0.02 ^b^	2600 ± 65.68 ^c^	1500 ± 24.26 ^b^	4100 ± 89.94 ^b^
T1	1.43 ± 0.14 ^ab^	0.90 ± 0.01 ^c^	0.25 ± 0.01 ^b^	9.29 ± 1.05 ^a^	5350 ± 70.71 ^ab^	2400 ± 53.55 ^a^	7750 ± 82.84 ^a^
T2	1.76 ± 0.21 ^ab^	2.66 ± 0.47 ^a^	0.66 ± 0.04 ^a^	9.17 ± 0.19 ^a^	5100 ± 82.84 ^ab^	2250 ± 60.06 ^ab^	7125 ± 76.77 ^a^
T3	1.21 ± 0.13 ^b^	2.02 ± 0.19 ^ab^	0.56 ± 0.01 ^a^	4.72 ± 0.22 ^b^	5550 ± 70.70 ^a^	2725 ± 76.77 ^a^	8275 ± 47.48 ^a^
T4	1.39 ± 0.20 ^ab^	1.53 ± 0.28 ^bc^	0.33 ± 0.00 ^b^	4.20 ± 0.37 ^b^	4500 ± 65.68 ^b^	2425 ± 88.90 ^a^	6925 ± 54.59 ^a^
*p* Value	0.014	0.001	0.013	0.02	0.021	0.00	0.001

Note: Data are presented as mean ± SD. The same column means with different superscripts differ significantly (*p* < 0.05).

**Table 4:** Effect of sex (female and male) and different nano-Se dosages on liver function indicators, immune response markers, oxidative stress, antioxidant levels, and enzyme activity for Nile tilapia *Oreochromis niloticus*.

Treatment	Gender	ALT ^1^	AST ^2^	LYS ^3^	IgM ^4^	GSH ^5^	LZM ^6^
C	M	41.1 ± 0.14 ^a^	41.15 ± 0.21 ^a^	444 ± 4.41 ^b^	1.95 ± 0.04 ^e^	18.9 ± 1.21 ^b^	27.5 ± 0.071 ^e^
T1	M	36.9 ± 0.14 ^b^	31.75 ± 0.35 ^b^	510 ± 5.42 ^a^	3.3 ± 0.2 ^d^	17.1 ± 1.14 ^c^	35.1 ± 2.02 ^c^
T2	M	32.8 ± 0.28 ^c^	31.9 ± 0.14 ^b^	339 ± 3.49 ^e^	3.6 ± 0.07 ^c^	16.1 ± 0.77 ^d^	29.93 ± 3.09 ^d^
T3	M	22.95 ± 0.07 ^d^	29.9 ± 0.14 ^c^	397.6 ± 4.71 ^c^	3.89 ± 0.01 ^a^	18.46 ± 0.66 ^b^	44.1 ± 0.14 ^b^
T4	M	20.77 ± 0.35 ^e^	27.84 ± 0.22 ^d^	369.6 ± 5.72 ^d^	3.79 ± 0.007 ^b^	19.2 ± 0.45 ^a^	51.96 ± 0.43 ^a^
*p* Value		0.01	0.001	0.02	0.01	0.05	0.002
C	F	36 ± 0.07 ^a^	40 ± 0.12 ^a^	271 ± 0.71 ^d^	2.55 ± 0.07 ^c^	14.26 ± 0.42 ^e^	25 ± 0.4 ^e^
T1	F	29.6 ± 0.85 ^b^	35 ± 0.14 ^b^	261 ± 1.14 ^e^	2.6 ± 0.3 ^c^	16.2 ± 0.07 ^c^	28.5 ± 0.7 ^d^
T2	F	22.83 ± 0.27 ^c^	29.5 ± 0.71 ^c^	332.5 ± 0.65 ^b^	3.2 ± 0.56 ^bc^	15.3 ± 0.06 ^d^	35.5 ± 0.72 ^c^
T3	F	20.5 ± 0.70 ^d^	27.5 ± 0.7 ^d^	313.5 ± 0.74 ^c^	3.8 ± 0.14 ^a^	19.1 ± 0.21 ^b^	41.84 ± 0.22 ^a^
T4	F	20.5 ± 0.72 ^e^	25.2 ± 0.28 ^e^	412.3 ± 0.49 ^a^	3.4 ± 0.07 ^b^	21 ± 0.14 ^a^	40.25 ± 0.35 ^b^
*p* Value		0.02	0.001	0.004	0.002	0.001	0.004

Note: Data are presented as mean ± SD. The same column with different superscripts differ significantly (*p* < 0.05). Treatments of males were compared with each other and treatments of females were compared with each other. ^1^ ALT: alanine aminotransferase; ^2^ AST: aspartate aminotransferase; ^3^ LYS: lysosome; ^4^ IgM: immunoglobulin M; ^5^ GSH: glutathione; ^6^ LZM: lysozyme. Data are presented as means.

## Data Availability

The original contributions presented in this study are included in the article. Further inquiries can be directed to the corresponding authors.
